# Identification of tumor microenvironment-related signature for predicting prognosis and immunotherapy response in patients with bladder cancer

**DOI:** 10.3389/fgene.2022.923768

**Published:** 2022-09-06

**Authors:** Zhihao Yao, Henghui Zhang, Xuejun Zhang, Zhiyong Zhang, Jirong Jie, Kunfeng Xie, Fei Li, Wanlong Tan

**Affiliations:** Department of Urology, Nanfang Hospital, Southern Medical University, Guangzhou, China

**Keywords:** bladder cancer, tumor microenvironment, risk model, prognosis, immunotherapy response

## Abstract

The tumor microenvironment (TME) not only provides fertile soil for tumor growth and development but also widely involves immune evasion as well as the resistance towards therapeutic response. Accumulating interest has been attracted from the biological function of TME to its effects on patient outcomes and treatment efficacy. However, the relationship between the TME-related gene expression profiles and the prognosis of bladder cancer (BLCA) remains unclear. The TME-related genes expression data of BLCA were collected from The Cancer Genome Atlas (TCGA) database. NFM algorithm was used to identify the distinct molecular pattern based on the significantly different TME-related genes. LASSO regression and Cox regression analyses were conducted to identify TME-related gene markers related to the prognosis of BLCA and to establish a prognostic model. The predictive efficacy of the risk model was verified through integrated bioinformatics analyses. Herein, 10 TME-related genes (PFKFB4, P4HB, OR2B6, OCIAD2, OAS1, KCNJ15, AHNAK, RAC3, EMP1, and PRKY) were identified to construct the prognostic model. The established risk scores were able to predict outcomes at 1, 3, and 5 years with greater accuracy than previously known models. Moreover, the risk score was closely associated with immune cell infiltration and the immunoregulatory genes including T cell exhaustion markers. Notably, the predictive power of the model in immunotherapy sensitivity was verified when it was applied to patients with metastatic urothelial carcinoma (mUC) undergoing immunotherapy. In conclusion, TME risk score can function as an independent prognostic biomarker and a predictor for evaluating immunotherapy response in BLCA patients, which provides recommendations for improving patients’ response to immunotherapy and promoting personalized tumor immunotherapy in the future.

## Introduction

Bladder cancer (BLCA) is the most diagnosed malignant tumor second only to the prostate in the genitourinary system, and the 11th most common tumor throughout the world (Witjes et al., 2021). There have been around 573,000 new cases diagnosed and 213,000 deaths of BLCA worldwide in 2020 (Sung et al., 2021). The main histologic type of BLCA is urothelial carcinoma (UC), which constitutes more than 90% of all bladder cancers (Dick et al., 2020). According to the invasion depth of the bladder wall, BLCA can be divided into non-muscle invasive bladder cancer (NMIBC) and muscle-invasive bladder cancer (MIBC). Over 70% of newly diagnosed BLCA are NMIBC, with a high rate of recurrence but low mortality (Kamat et al., 2016). However, up to 20–25% of patients are identified at initial diagnosis as MIBC, accounting for the cause of most deaths from BLCA (Knowles and Hurst, 2015). Half of MIBC patients develop distant metastasis within 2 years, and 60% die within 5 years, despite taking aggressive treatment (Stein et al., 2001; Madersbacher et al., 2003; Shariat et al., 2006) Currently, cisplatin-based chemotherapy remains the standard first-line treatment for patients with inoperable locally advanced or metastatic urothelial cancer (Witjes et al., 2021). Unfortunately, almost all patients will eventually progress and die from BLCA (Lopez-Beltran et al., 2021). Considering the high mutational burden of BLCA, immunotherapy has become an alternative treatment for patients with advanced or metastatic UC, especially immune checkpoint inhibitors (ICIs). Although ICIs targeting the programmed cell death-1 (PD-1)/programmed death ligand-1 (PD-L1) axis show a promising result in the treatment of UC (Sharma et al., 2017b), the rate of partial or complete response to ICI therapy is only 20–30% in UC patients (Mariathasan et al., 2018). Thus, it is urgently needed to identify reliable prognostic biomarkers that can predict clinical outcomes and guide targeted clinical therapy, so as to improve the prognosis and increase the proportion of responders to immunotherapy in BLCA.

Recent studies have revealed the significance of the tumor microenvironment (TME) in tumorigenesis, immune evasion, and therapeutic response (Casey et al., 2015; Koi and Carethers, 2017; Wu and Dai, 2017; Hinshaw and Shevde, 2019). TME is mainly composed of immune infiltrating cells, tumor-related fibroblasts, blood vessels, as well as extracellular matrix (ECM) and signaling molecules (Tibshirani, 2011; Kulbe et al., 2012). Tumor cells develop in complex and dynamic TME, which forms an immunosuppressive microenvironment and leads to resistance to chemotherapy and targeted therapy (Whiteside, 2008; Hui and Chen, 2015). Notably, studies have shown that the degree and proportion of tumor-infiltrating cells contribute to the distinct prognosis of BLCA patients. Immune cells with immunosuppressive phenotypes such as myeloid-derived suppressor cells (MDSCs), tolerogenic DCs (tDCs), tumor-associated macrophages (TAMs), and regulatory T cells (T regs) accumulate in BLCA (Crispen and Kusmartsev, 2020). Inflammatory cancer-associated fibroblasts (iCAFs) have proliferative properties and are significantly associated with a poor prognosis of BLCA (Chen et al., 2020). The proportion of CD8^+^ T cells is significantly associated with the tumor stage of BLCA and decreases with increasing tumor stage. The increased level of CD8^+^ T cells leads to a longer survival time (Wang et al., 2020), implying that infiltration of immune cells affects the prognosis of BLCA and may help develop the method to evaluate the immunotherapy response.

Taken together, these results indicate that acquiring a better understanding of TME and clarifying the underlying mechanism is of great significance in evaluating the prognosis and guiding the treatment of BLCA patients. Consequently, in this study, we aimed to design a risk signature based on TME-related genes to predict the response to immunotherapy in BLCA patients.

## Materials and methods

### Data collection and preprocessing

The RNA-seq (FPKM values) and somatic mutation data (MuTect2) of BLCA patients were obtained from the TCGA data portal (https://portal.gdc.cancer.gov/) and the FPKM values were transformed into TPM values. The clinicopathological characteristics of the TCGA-BLCA samples were curated from the cBioportal for Cancer Genomics database (https://www.cbioportal.org/). The microarray mRNA expression profile of GSE13507 was downloaded from the Gene Expression Omnibus (GEO) database (https://www.ncbi.nlm.nih.gov/geo/), which was used as the external validation cohort to verify the reliability of the TCGA data. The IMvigor210 cohort consisting of mUC patients treated by ICIs, was obtained through the R package “IMvigor210CoreBiologies” and was used to predict the immunotherapy response in BLCA patients. TME-related genes were obtained from previous studies (Newman et al., 2015; Rooney et al., 2015; Becht et al., 2016; Chifman et al., 2016; Li et al., 2016; Tirosh et al., 2016; Aran et al., 2017), which, after summarizing, provided 4,061 genes (Supplementary Table S1).

### Identification of TME-related differentially expressed genes

Based on the expression level of the TME-related genes in TCGA-BLCA cohort, the R package “limma” was used to identify the differentially expressed genes (DEGs) between tumor tissues and normal tissues (Ritchie et al., 2015), with criterion set as the absolute value of log2fold change (|log2FC|) >1 and false discovery rate (FDR) < 0.05. Heatmap and volcano plots of DEGs were constructed using the “pheatmap” and “ggplot2” R package.

### Consensus molecular clustering of the TME-related DEGs by NMF

A total of 1018 TME-related DEGs were gained and a univariate Cox analysis was performed to select prognostic genes of TCGA-BLCA cohort (*p* < 0.01). The consensus clustering method was applied to classify patients into distinct molecular patterns according to the prognostic gene expression matrix. The R package “NMF” under the method of the brunet algorithm, with nruns setting at 100 and rank from 2 to 10, was conducted to determine the number of clusters and guarantee the stability of clustering (Gaujoux and Seoighe, 2010).

### Estimation of immune cell infiltration

The deconvolution approach- CIBERSORT (http://cibersort.stanford.edu/), a widely used method of characterizing the cell composition of complex tissues via an LM22 gene signature matrix (Newman et al., 2015), was applied to quantify the relative proportion of 22 distinct immune cell types in the tumor microenvironment of TCGA-BLCA samples. The sum of relative percentages of immune cells in each tumor sample is equal to 1. The results of immune cell infiltration were obtained through the “CIBERSORT R script v1.03” (CIBERSORT.R).

### Construction of the prognostic TME-related gene signature

The expression data of TME-related DEGs from the TCGA-BLCA cohort was used in the process of model training. Firstly, 70% of the TCGA-BLCA samples were randomly selected as the train cohort, and the remaining 30% were selected as the internal test cohort. The R package “survival” was used to perform the univariate Cox regression analysis to select the candidate genes that were significantly related to the prognosis of the train cohort. Based on the R package “glmnet”, LASSO Cox regression analysis was performed to minimize the number of the candidate genes and to avoid overfitting of the model (Friedman et al., 2010; Tibshirani, 2011). The genes selected by LASSO regression analysis were under further analysis. Finally, the candidate genes above were subjected to multivariate Cox regression analysis and screened out 10 TME-related genes that construct the best prognostic gene signature. The risk score of each sample was calculated with the optimized genes based on the following formula: $\text{Risk}\;\text{score}=\overset{n}{\underset{i=1}{\sum }}Coef_{i}\text{*}X_{i}$ , where $Coef_{i}$ is the risk coefficient of each factor calculated by the multivariate Cox model, and $X_{i}$ is the expression level of each TME-related genes.

### Establishment and assessment of the nomogram

Univariate and multivariate Cox regression analyses were also used to calculate the hazard ratios and pick out independent prognostic factors. The nomogram combining the significant factors was plotted using R package “rms” (Zhang and Kattan, 2017). Calibration curve was plotted to evaluate the performance of the nomogram (Vickers et al., 2019). Moreover, decision curve analysis (DCA) was employed to measure the clinical utility of the nomogram compared to different decision strategies (Vickers and Holland, 2021). The *x*-axis indicates the percentage of threshold probability, and the *y*-axis represents the net benefit.

### Comparison of different BLCA prognostic models

Besides comparing the prognostic value of the TME-related gene signature with other clinical parameters, we also downloaded four prognostic gene signatures of BLCA from the previous literature to demonstrate that the model had a better clinical utility. Then, the concordance index (C-index) and restricted mean survival (RMS) curve were used for the estimation of our model compared with the other gene signatures.

### Gene Set Enrichment Analysis

GSEA was performed to explore different pathways between the high and low-risk groups. Gene ontology gene sets “c2. cp.kegg.v7.4. symbols.gmt” were downloaded from Molecular Signatures Database (MSigDB, https://www.gsea-msigdb.org/gsea/msigdb/index.jsp) for enrichment analysis. The enriched gene sets with *p*-value less than 0.05 were considered to be statistically significant.

### Prediction of immunotherapy sensitivity

The immunophenoscore (IPS) of TCGA-BLCA was downloaded from The Cancer Immunome Atlas (TCIA, https://tcia.at/home). The TIDE score of each sample was calculated after submitting the normalized expression data to the website of tumor immune dysfunction and exclusion (TIDE, http://tide.dfci.harvard.edu/). The immunotherapy response of patients could be inferred by the TIDE score and IPS. In general, a higher IPS and lower TIDE score are considered as a better response to immunotherapy. The number of predicted neoantigens of TCGA-BLCA samples was gained from a published article (Rooney et al., 2015). Moreover, an independent anti-PD-L1 immunotherapy cohort, IMvigor210 was applied to confirm the predictive value of the TME-related gene signature.

### Statistical analysis

The statistical analyses in our study were performed using R (version 4.1.0) software. Statistical significance was set at a probability value of *p*-value<0.05. For comparisons of the two groups, Wilcoxon test was used as a nonparametric method. Spearman’s correlation test was applied to evaluate relationships between two variables. The Kaplan-Meier survival curves were built to evaluate survival differences based on the R package “Survminer”. The receiver operating characteristic (ROC) curve was generated to assess the prognosis classification performance of the TME-related model, and the area under the curve (AUC) was calculated using R package “timeROC”. The C-index and RMS cure were performed using the “survival” and “survcomp” packages. In terms of each test, a *p*-value <0.05 suggested a significant difference. *p-value <0.05, **p-value <0.01, and ***p-value <0.001 express statistically significant characteristics.

## Results

### Identification of TME-related DEGs in TCGA-BLCA

The flow chart describing our study is shown in Supplementary Figure S1. A total of 1018 TME-related genes were identified as DEGs in tumor tissues when compared with normal tissues (Supplementary Table S2). The heatmap and volcano plots were made (Supplementary Figures S2A, B). Among the TME-related DEGs, 773 genes were found to be significantly up-regulated, while 245 genes were found to be significantly down-regulated.

### Recognition of different molecular clusters mediated by TME-related genes

**FIGURE 1:**
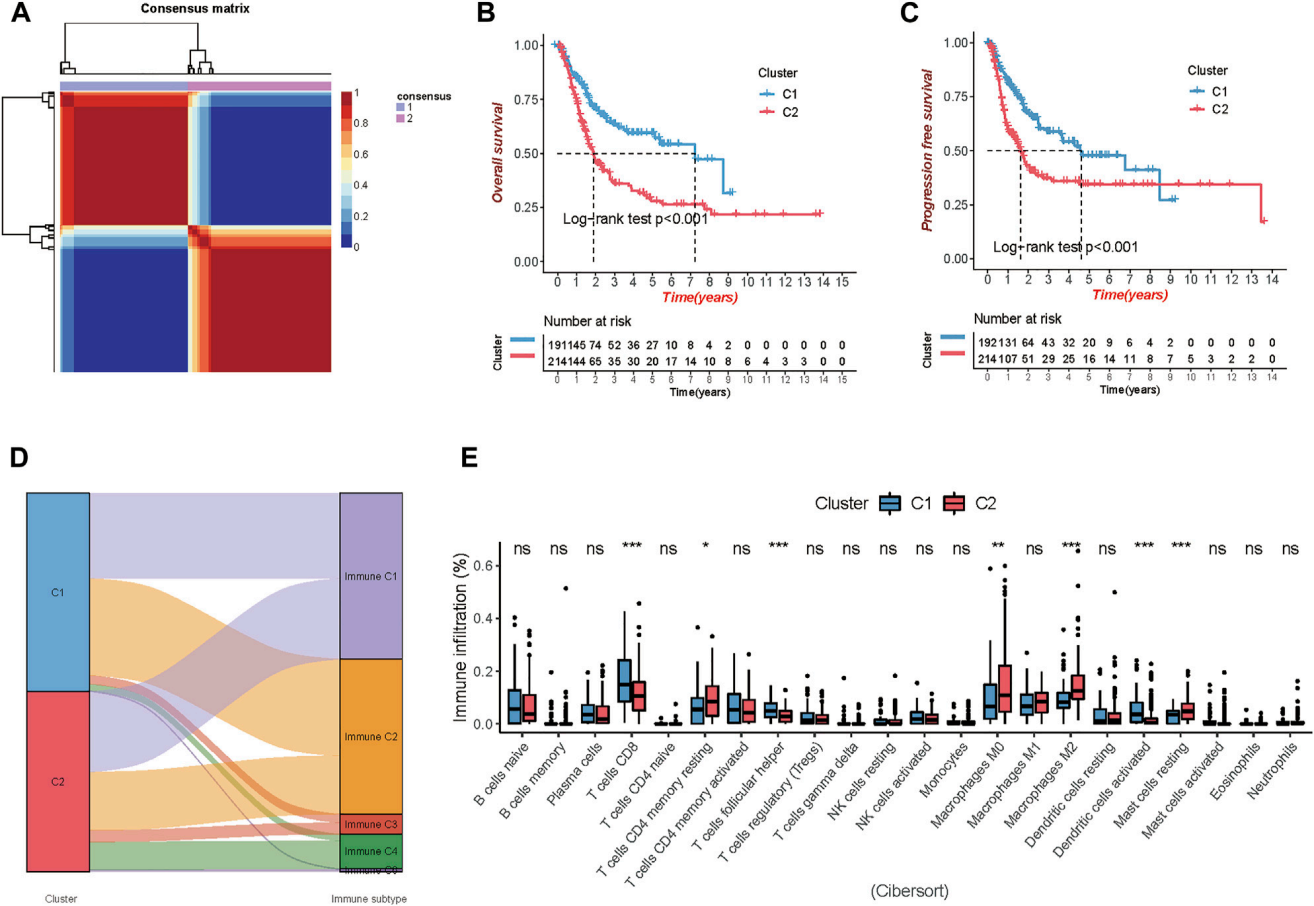
Comparison of the two clusters 1 and 2 (C1 and C2) in TCGA-BLCA cohort. **(A)** Consensus map clustered via the non-negative matrix factorization (NMF) algorithm. **(B,C)** Overall survival (OS) and progression-free survival (PFS) showed significant differences. **(D)** Percentage of the immune subtypes accounting for each of the two clusters. **(E)** The fraction of tumor-infiltrating immune cells in the two clusters using the CIBERSORT algorithm (*p < 0.05; **p < 0.01; ***p < 0.001).

In the expression matrix of the whole TCGA-BLCA samples with 1,018 genes, 105 prognostic TME-related genes were obtained by univariate Cox analysis. We included the prognostic genes to stratify samples with different TME clustering properties using consensus clustering analysis of the NMF algorithm. As shown in Supplementary Figures S3, S4, two clusters could achieve the best clustering efficacy. Accordingly, we identified two distinct molecular patterns, involving 191 cases in cluster 1 and 214 cases in cluster 2 (Figure 1A). Between the two clusters, cluster 1 exhibited a prominent survival advantage in OS and PFS, whereas cluster 2 had a bad prognosis in the TCGA-BLCA cohort (log-rank test, both of *p* < 0.001; Figures 1B,C).

Additionally, immune subtypes (C1-C6) based on the transcriptomic profiles of TCGA database, a new global immune classification of solid tumors (Thorsson et al., 2019), were used to observe the subtype distribution between the above two clusters. The alluvial diagram showed the distribution of cases between immune subtypes and two clusters (Figure 1D). To visualize the immune cell infiltration in the TME, we compared the TME composition of two distinct TME clusters to clarify the differences between the two clusters. The infiltration levels of CD8^+^ T cells, follicular helper T cells, and activated dendritic cells were significantly higher in cluster 1. Patients in cluster 1 were characterized by a significantly higher infiltration level of resting memory CD4^+^ T cells, non-activated Macrophages (M0) and anti-inflammatory Macrophages (M2), and resting mast cells (Figure 1E).

### Establishment of the prognostic TME-related gene signature

**FIGURE 2:**
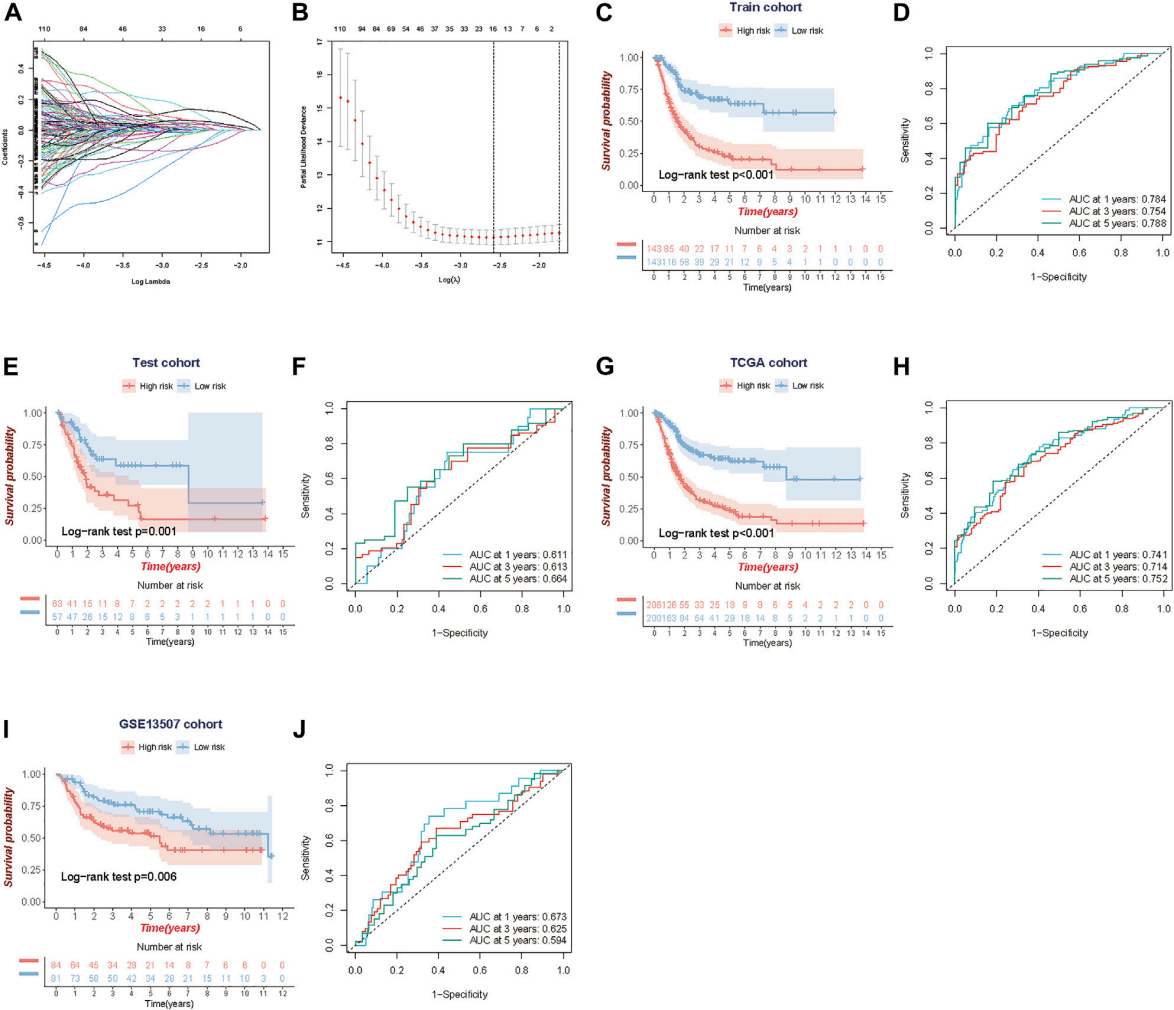
Construction and validation of the TME-related 10-gene risk score (RS). **(A,B)** LASSO regression analysis and partial likelihood deviance on the prognostic genes. **(C, D)** Kaplan-Meier survival curves and ROC curve for TCGA train cohort. **(E,F)** Kaplan-Meier survival curves and the ROC curve for TCGA test cohort. **(G, H)** Kaplan-Meier survival curves and ROC curve for the whole TCGA cohort. **(I,J)** Kaplan-Meier survival curves and ROC curve for GSE13507 cohort.

The TCGA-BLCA samples were randomly divided into the train (70%, *n* = 286) and internal test (30%, *n* = 120) cohorts, and there were no significant differences in gender, age, histological subtype, tumor stage, grade, and event between the train and validation cohorts (Table 1). We applied LASSO and Cox regression analyses to further narrow the range of prognostic genes, and finally, constructed a TME-related gene signature involving 10 genes in the train cohort. We also analyzed the whole landscape of genetic alterations of these 10 TME-related genes, including the CNV variation frequency (Supplementary Figure S5A), the gene mutation frequency (Supplementary Figure S5B, and the correlation analysis between the TME-related genes expression and DNA methylation level (Supplementary Figures S5C–K). The results of LASSO analysis were shown in Figures 2A,B. Risk score for each patient was calculated as follows:

$$\begin{array}{l} risk\;score=PFKFB4\;expression*0.156+P4HB\;expression*0.370+\\ OR2B6\;expression*\left(-0.528\right)+OCIAD2\;expression*\left(-0.298\right)+\\ OAS1\;expression*\left(-0.226\right)+KCNJ15\;expression*\left(-0.105\right)+\\ AHNAK\;expression*0.210+RAC3\;expression*0.223+\\ EMP1\;expression*0.150+PRKY\;expression*\left(-0.141\right). \end{array}$$

**TABLE 1:** The clinical characteristics of the train cohort and validation cohort.

Clinical characteristics		Train cohort	Validation cohort	*p*-value
Gender	Female	73 (25.52%)	33 (27.5%)	0.772
	Male	213 (74.48%)	87 (72.5%)	
Age	≤65	113 (39.51%)	47 (39.17%)	1
	>65	173 (60.49%)	73 (60.83%)	
Histological subtype	Non-Papillary	193 (67.48%)	78 (65%)	0.3141
	Papillary	91 (31.82%)	39 (32.5%)	
	unknown	2 (0.7%)	3 (2.5%)	
Tumor stage	Stage I	0 (0%)	2 (1.67%)	0.211
	Stage II	90 (31.47%)	39 (32.5%)	
	Stage III	101 (35.31%)	39 (32.5%)	
	Stage IV	93 (32.52%)	40 (33.33%)	
	unknown	2 (0.7%)	0 (0%)	
Grade	High Grade	269 (94.06%)	113 (94.17%)	0.9849
	Low Grade	15 (5.24%)	6 (5%)	
	unknown	2 (0.7%)	1 (0.83%)	
Event	Censored	161 (56.29%)	67 (55.83%)	1
	Dead	125 (43.71%)	53 (44.17%)	

Using the median risk score in the train cohort as the cutoff point, the patients were divided into low-risk and high-risk groups. KM survival analysis indicated that the low-risk group had better overall survival than the high-risk group (log-rank test, *p* < 0.001) (Figure 2C), and time-dependent ROC analysis showed that the predictive accuracy of the TME-related gene signature was 0.784 at 1 year, 0.754 at 3 years and 0.788 at 5 years in the train cohort (Figure 2D). We further assess the predictive value of the TME-related gene signature in the internal test cohort, the whole TCGA cohort, and an independent external cohort. The results from the above data (test cohort, TCGA cohort and GSE13507 cohort) shared the same trend in survival, with great significance (log-rank test, *p* = 0.001, *p* < 0.001 and *p* = 0.006), and the AUC was 0.611, 0.741, and 0.673 at 1 year; 0.613, 0.714, and 0.625 at 3 years; and 0.664, 0.752 and 0.594 at 5 years, respectively (Figures 2E–J). Meanwhile, we also performed the Kaplan-Meier survival analyses for patients with different clinical characteristics in TCGA-BLCA cohort (Supplementary Figures S6A–F). By comparing risk scores between different clinical characteristics, we found that some clinical factors indicating poor prognosis were strongly associated with a higher risk score, including older age, more advanced tumor stage, and so on (Supplementary Figures S7A–H).

### Establishment and assessment of the nomogram

**FIGURE 3:**
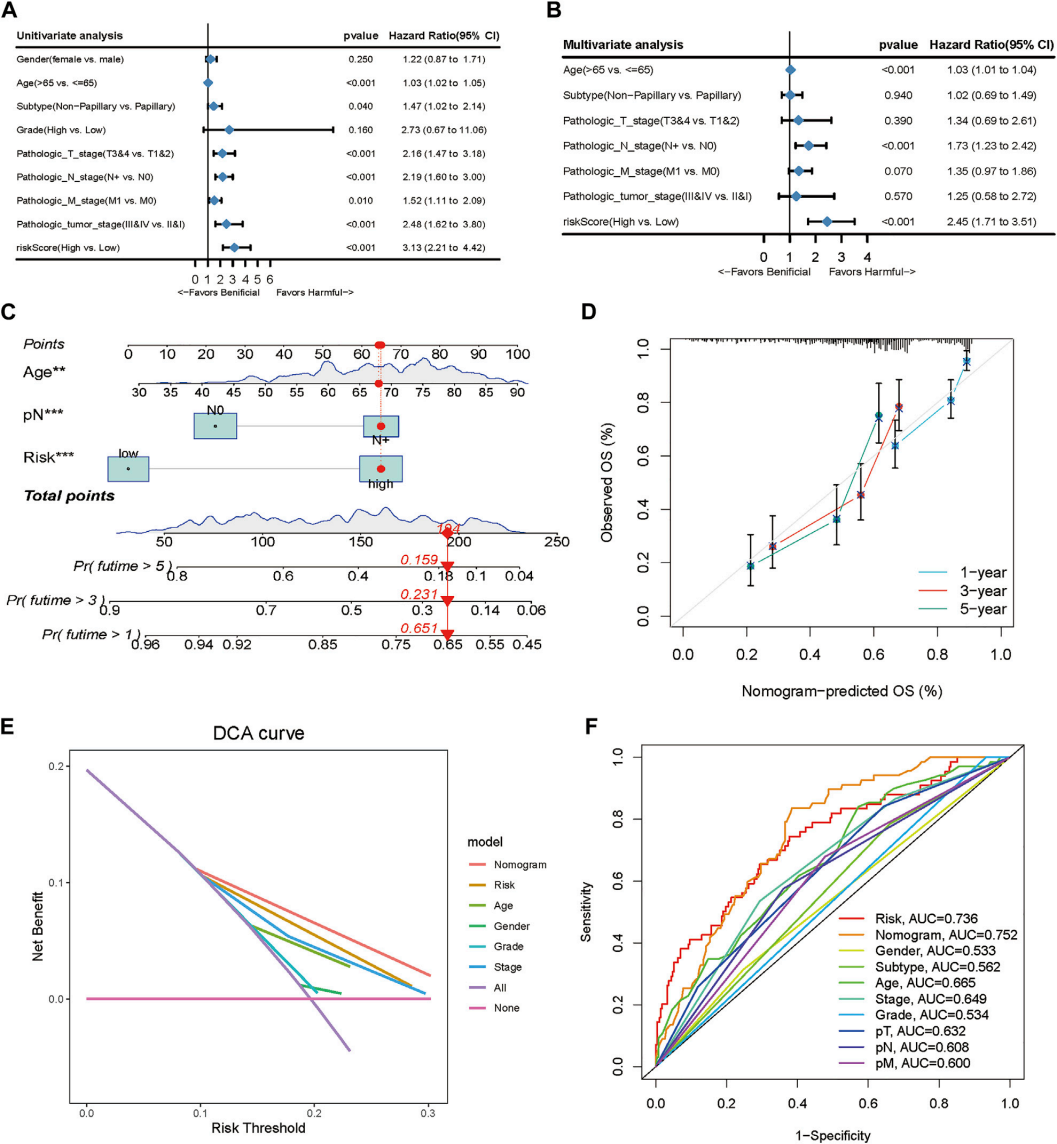
Clinical value of the TME-related prognostic model. **(A, B)** Univariate and multivariate Cox regression applied to analyze the relationship between the risk score and clinical prognosis. **(C)** Nomogram predicting the 3- and 5-year OS for patients. **(D)** Calibration curves for the nomogram predicted 3- and 5-year OS for patients in relation to actual survival. **(E)** Decision curve analysis (DCA) curves evaluated the clinical benefit of the nomograms and their potential scope of application. **(F)** ROC curves of the nomograms compared with other clinical variables.

To validate the predictive efficiency of the gene signature in TCGA-BLCA cohort, we included risk score and several clinicopathological characteristics into the univariate and multivariate analyses. Univariate Cox regression analysis indicated that age (HR = 1.03, 95%CI 1.02–1.05, *p* < 0.001), subtype (HR = 1.47, 95%CI 1.02–2.14, *p* < 0.001), T (HR = 2.16, 95%CI 1.47–3.18, *p* < 0.001), N (HR = 2.19, 95%CI 1.60–3.00, *p* < 0.001), M (HR = 1.52, 95%CI 1.11–2.09, *p* = 0.01), stage (HR = 2.48, 95%CI 1.62–3.8, *p* < 0.001) and risk score (HR = 2.45, 95%CI 1.71–3.51, *p* < 0.001) were significantly correlated with OS (Figure 3A). Subsequent multivariate Cox regression analyses showed that age (HR = 1.03, 95%CI 1.01–1.04, *p* < 0.001), N (HR = 1.73, 95%CI 1.23–2.42, *p* < 0.001) and risk score (HR = 2.45, 95%CI 1.71–3.51, *p* < 0.001) were still confirmed as independent factors for OS (Figure 3B). Next, we developed a nomogram based on the above 3 variables to improve the clinical practicability of the model (Figure 3C). The calibration graph of nomogram exhibited a high consistency between the observed probability of 1-,3- and 5-year OS and the nomogram-predicted probability (Figure 3D). Furthermore, using the decision curve (DCA), we found that nomogram had higher predictive efficiency for OS than other relevant clinical parameters (Figure 3E). Meanwhile, multivariable ROC curve showed that the AUC of the nomogram was the highest compared with others (AUC = 0.752) (Figure 3F).

### Comparison of different BLCA prognostic models

**FIGURE 4:**
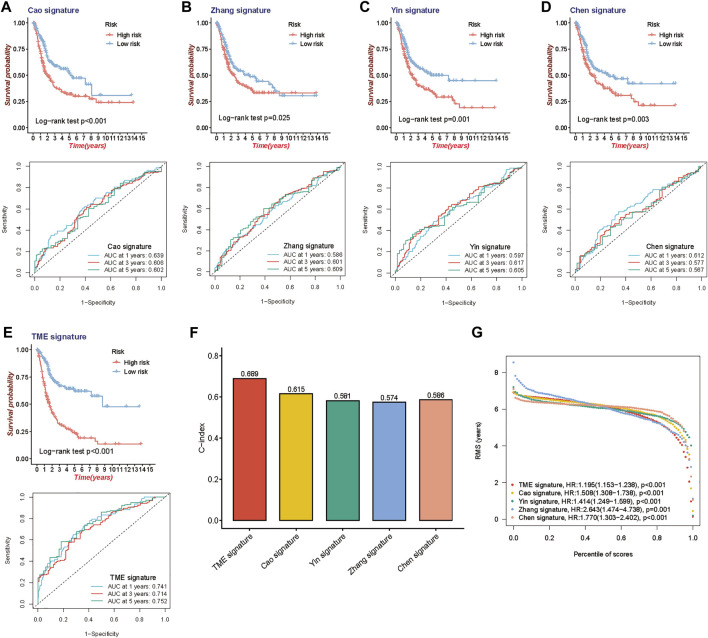
Comparison of the 10-gene risk model with other established models. **(A–E)** Kaplan-Meier survival curves and ROC curve of TME gene signature with four other published gene signatures. **(F)** Concordance index (C-index) of the five prognostic risk models. **(G)** Restricted mean survival (RMS) time curve of the five prognostic risk models.

To further assess the survival classification and predictive performance of the model, we not only compared the prognostic value of the TME-related gene signature with other clinical parameters but also compared the predictive efficiency of four published prognostic models of BLCA. Cao signature was an EMT-related gene signature for BLCA (Cao et al., 2020); Zhang signature was a glycolysis-based gene signature (Zhang et al., 2020); Yin signature was a gene signature consisting of 13 mRNAs (Yin et al., 2020), and Chen signature was a gene signature derived from microarray data of BLCA (Chen et al., 2019). Both KM survival method and ROC curve were used to observe the predictive performance of the above gene signatures. The results of KM survival curves from the above gene signature (TME signature, Cao signature, Zhang signature, Yin signature and Chen signature) had the same trend in survival, with great significance (log-rank test, *p* < 0.001, *p* < 0.001, *p* = 0.025 and *p* = 0.003), and the AUC was 0.741, 0.639, 0.586, 0.597 and 0.612 at 1 year; 0.714, 0.606, 0.601, 0.617 and 0.577 at 3 years; and 0.752, 0.602, 0.609, 0.605 and 0.567 at 5 years, respectively (Figures 4A–E). When quantifying the predictive accuracy of each gene signature, it was evident that the C-index for TME signature (C-index = 0.689), as well as the RMS survival curves, were more favorable than others (C-index 0.615, 0.581, 0.574, and 0.586 for Cao signature, Yin signature, Zhang signature, and Chen signature) (Figures 4F,G).

### Gene set enrichment analysis between high and low-risk groups

**FIGURE 5:**
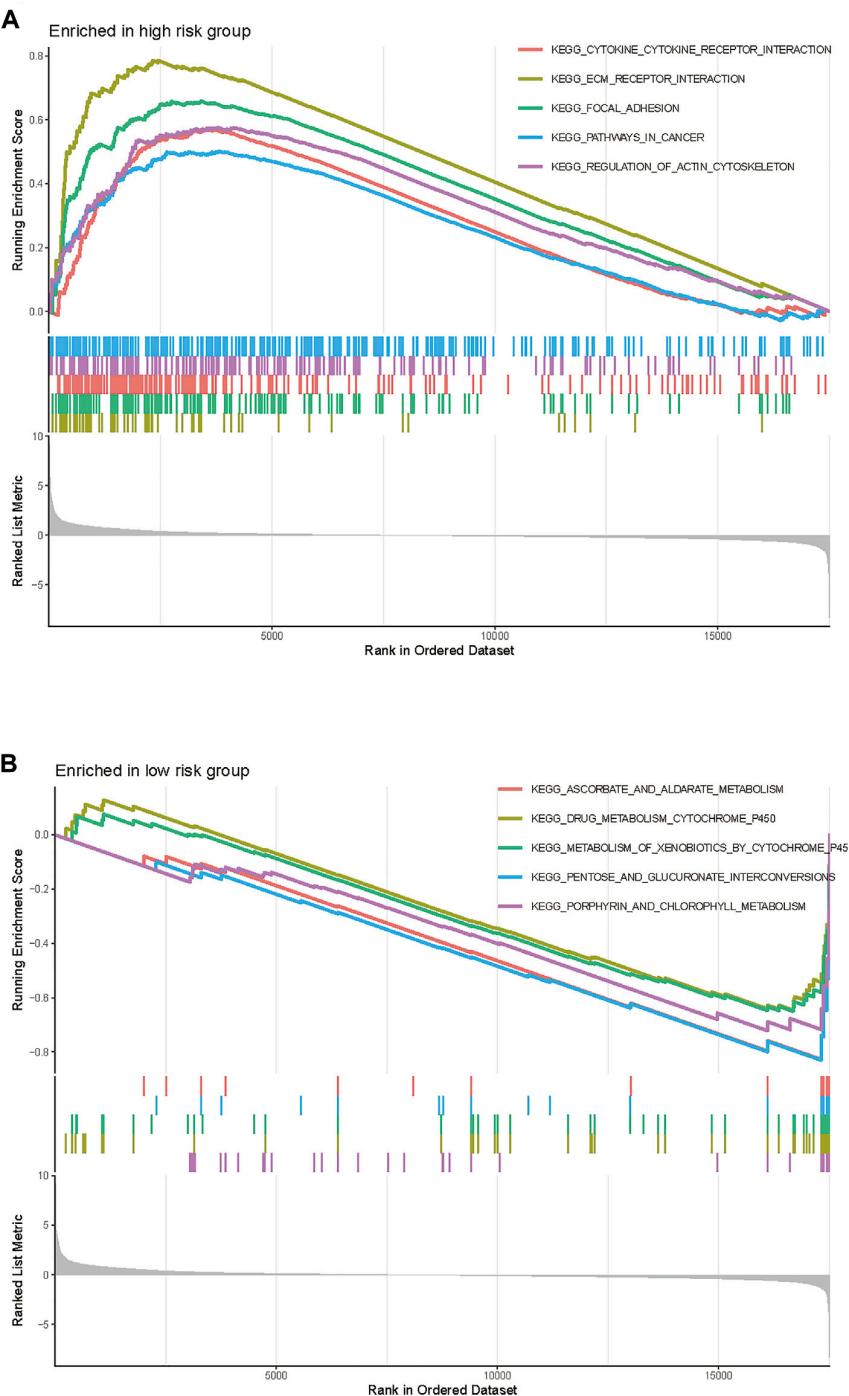
Gene Set Enrichment Analysis of the TME gene signature in TCGA-BLCA cohort. **(A)** The enriched gene sets in high-risk group (nominal *p* < 0.05). **(B)** The enriched gene sets in low-risk group (nominal *p* < 0.05).

GSEA was conducted to explore the underlying biological process between the high-risk and low-risk patients stratified by the TME signature. We found that the top five KEGG pathways including cytokine-cytokine receptor interaction, ECM receptor interaction, focal adhesion, pathways in cancer, and regulation of actin cytoskeleton were significantly increased in the high-risk group (Figure 5A), while the ascorbate and aldarate metabolism, drug_metabolism_cytochrome_P450, metabolism of xenobiotics by cytochrome P450, pentose and glucuronate interconversions and porphyrin and chlorophyll metabolism were significantly increased in the low-risk group (Figure 5B).

### The relationship of the TME-related gene signature with immune cells infiltration and immunoregulatory genes

**FIGURE 6:**
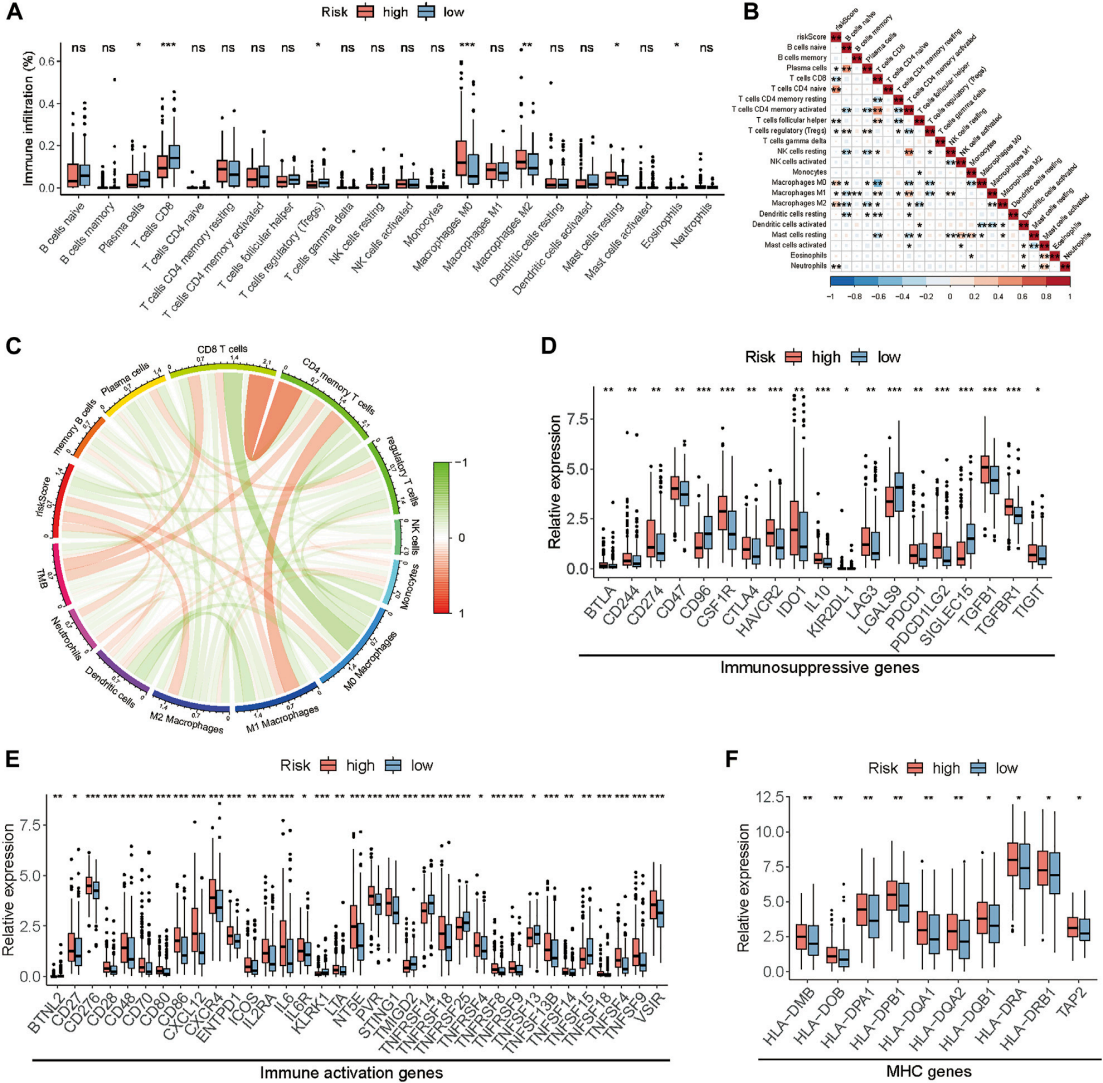
The relationship between the risk score and the TME in TCGA-BLCA cohort. **(A)** The fraction of tumor-infiltrating immune cells between the high and low-risk groups. **(B)** The correlation analysis between risk score and tumor-infiltrating immune cells. **(C)** The correlation analysis among risk score, immune cells, and TMB. **(D–F)** The differences in the expression of immunoregulatory genes. (*p < 0.05, **p < 0.01, ***p < 0.001).

To explore the influence of the prognostic model on immune cells infiltration, we estimated the proportion of 22 types of infiltrating immune cells based on the CIBERSORT algorithm. Next, we compared the proportional differences of each type of immune cell between the high-risk and the low-risk group and investigated the association between risk score and immune cells infiltration. Samples in the low-risk group had a significant increase in the levels of CD8^+^ T cells and plasma cells and a significant decrease in the fraction of M0 macrophages and M2 macrophages (Figure 6A). Meanwhile, correlation analysis indicated that the risk score was negatively correlated with CD8^+^ T cells, plasma cells, follicular helper T cells, and M1 macrophages but positively correlated with CD4^+^ naïve T cells, M0 macrophages, and M2 macrophages (Figures 6B,C). Furthermore, we investigated the immunoregulatory genes expression between the high-risk and the low-risk group, including the gene sets of MHC, immunosuppression, and immune activation. Consequently, we found that most of the immunoregulatory genes, such as the T cell exhaustion markers (PDCD1/PD1, CTLA4, TIM3, LAG3, VSIR, and TIGIT), were highly expressed in the high-risk group compared to the low-risk group (Figures 6D–F). To explain the relationship between risk score and commonly used biomarkers for predicting immunotherapy response, correlation analyses were performed. The results showed that the risk score negatively correlated with MSI and positively correlated with some classical immune checkpoints, but all of these relationships were weak (Supplementary Figures S8A–I).

### Prediction of immunotherapy sensitivity *via* TME-related gene signature

**FIGURE 7:**
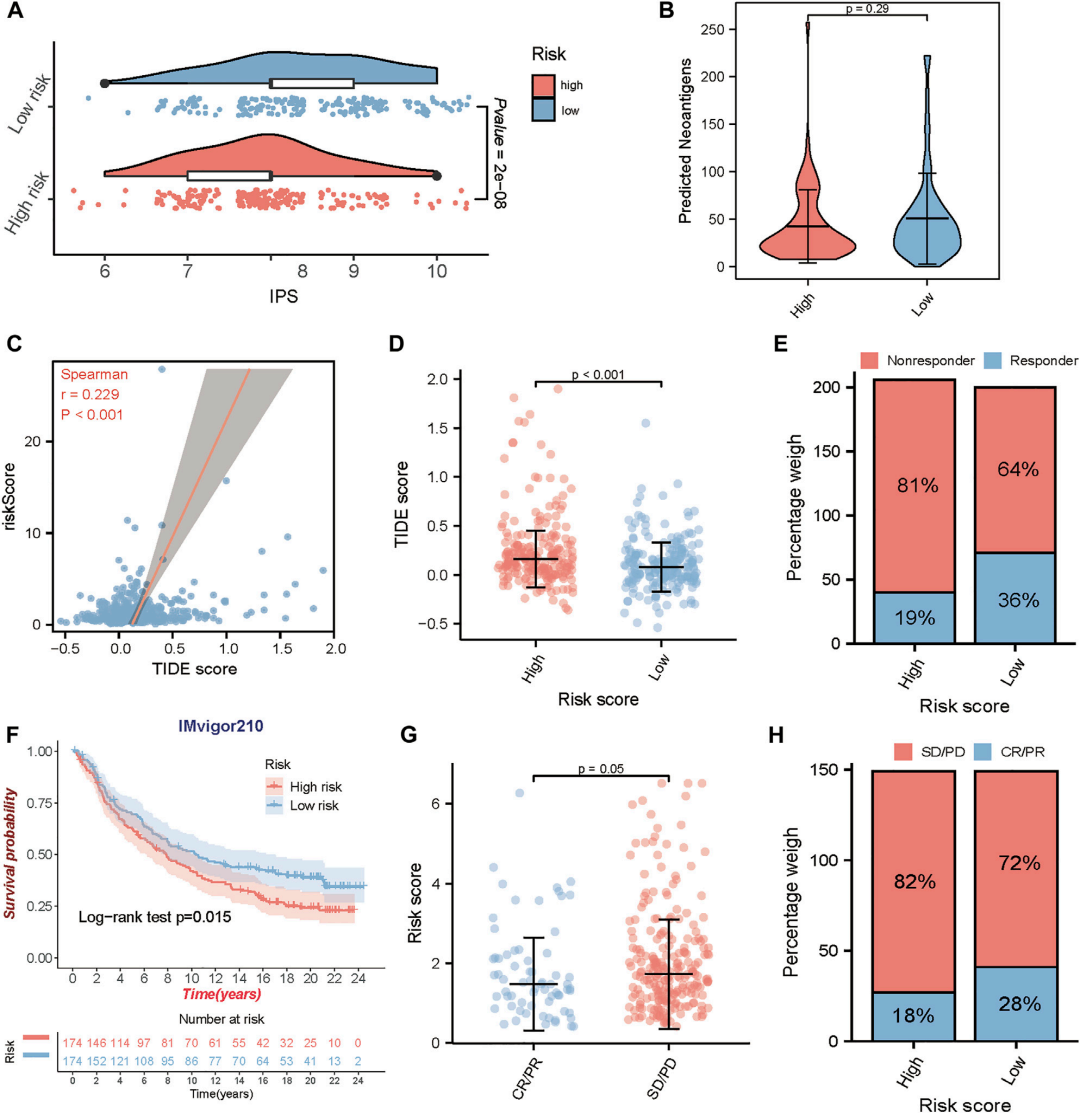
Prediction of immunotherapeutic benefits using the risk score. In TCGA-BLCA cohort, **(A)** the relative distribution of the immunophenoscore (IPS) between the high and low-risk groups. **(B)** The difference of the predicted neoantigens in the high and low-risk groups. **(C, D)** The correlation analysis between risk score and TIDE score, and the comparison of the TIDE score in the high and low-risk groups. **(E)** The proportion of patients with response to immunotherapy in high or low-risk groups. In IMvigor210 cohort, **(F)** Kaplan-Meier survival curves for high and low-risk groups. **(G)** The comparison of the risk score between mUC patients with different clinical responses (CR/PR and SD/PD). **(H)** The proportion of patients with clinical response to anti-PD-L1 immunotherapy in high or low-risk groups. CR, complete response; PR, partial response; SD, stable disease; PD, progressive disease.

Immune checkpoints are important regulators of the immune system, which maintain immune homeostasis and regulate immune responses. Immunotherapy, represented by PD-L1/PD-1 inhibitors, is of great value in cancer treatment and has become an important way in antitumor therapy recently. The newly identified indicators, such as IPS, TIDE score, and predicted neoantigens, were used to evaluate the possibility of immunotherapy response in TCGA-BLCA samples. Accordingly, we found that the IPS was significantly elevated in the low-risk group (Figure 7A), and TIDE score which positively correlated with the risk score (Figure 7C) was significantly decreased in the low-risk group (Figure 7D). Although there was no significant difference in the expression of predicted neoantigens between the two groups, the violin plot showed a trend that the expression of neoantigens increased in the low-risk group (Figure 7B). Meanwhile, we also found that patients in the low-risk group would be more likely to respond to immunotherapy compared to the high-risk group (response rate: 36 vs. 19%) (Figure 7E). These findings indirectly demonstrated that low-risk patients could benefit more from immunotherapy.

To determine whether the risk score could predict response to immunotherapy in BLCA, we further analyzed an anti-PD-L1 immunotherapy cohort- IMvigor210. Similarly, patients in IMvigor210 cohort were classified into the high-risk and the low-risk group by the median risk score. A consistent result was observed in IMvigor210 cohort, patients in the low-risk group had a significantly prolonged OS (log-rank test, *p* = 0.015) (Figure 7F), and patients with responses to anti-PD-L1 treatment exhibited lower risk score in the IMvigor210 cohort (*p* = 0.05) (Figure 7G). In addition, patients in the low-risk group exhibited a higher objective response rate (CR/PR) to anti-PD-L1 immunotherapy than those in the high-risk group (Figure 7H). These results strongly indicated that the TME risk score is significantly associated with anti-PD-L1 immunotherapy responses and can help to predict the anti-PD-L1 immunotherapy response in BLCA.

## Discussion

Although immunotherapy brings new hope to patients with advanced cancer, only 20–40% of patients respond to immunotherapy (Sharma et al., 2017a). Rather than only focusing on tumor cells themselves, a growing number of studies have turned their attention to the TME, an environment around tumor composed of immune components, blood vessels, extracellular matrix, and signaling molecules (Tsai et al., 2014). Growing evidence has shown that TME has a significant impact on the growth and development of cancerous cells, and affects the response to immunotherapy (Murciano-Goroff et al., 2020).

First, we included 413 BLCA samples and 4061 TME-related genes from TCGA database. Two molecular clusters were established by the NMF method, which has become a widely used tool for the analysis of high-dimensional data. Next, we compared the proportion of infiltrating immune cells between the two clusters. Previous studies have shown that dendritic cells (DCs) are antigen-presenting cells and responsible for the initiation of adaptive immune responses through interacting with T cells and B cells. As members of the immune system, T cells, composed of two main subtypes of CD4^+^ and CD8^+^ T cells, play a central role in the adaptive immune response. CD4^+^ T cells, also known as T helper cells, mediate the humoral immunity and can further differentiate into follicular helper T cells (Tfhs) and regulatory T cells (Tregs) after being activated. Tfhs are responsible for cooperating with B cells to promote and regulate humoral immune responses (Jia et al., 2015), and the infiltration of Tfhs can increase the survival of cancer patients (Nurieva et al., 2019). Tregs have the capacity to maintain the balance of immune responses; but in tumors, they suppress antitumor effects of immune cells, and thus are considered to correlate with tumor escape from the host immune system (Whiteside, 2014). CD8^+^ T cells, another subtype of T cells, play a crucial role in the host response to antitumor immunity through their cytotoxicity effect to kill tumor cells, and they are thought to be an important reason for the success of cancer immunotherapy (Raskov et al., 2021). In our study, cluster 1 with a better prognosis has higher infiltration levels of CD8^+^ T cells, follicular helper T cells, and activated dendritic cells (DCs). The results indicated that with the help of DCs, the infiltration of activated CD8^+^ T cells and Tfh can improve BLCA survival, which is consistent with previous studies (Shi et al., 2018; Winerdal et al., 2018). Macrophages, including M1 and M2 macrophages, are mainly involved in innate immunity and help initiate adaptive immunity (Hao et al., 2012). M1 macrophages, representing the antitumor activity, mainly produce proinflammatory factors to induce inflammation which leads to antitumor reaction. Whereas M2 macrophages, as another type of tumor-associated macrophages (TAMs), suppress the immune response by secreting inhibitory cytokines, thus promoting tumor progression (Chanmee et al., 2014; Lin et al., 2019). In line with previous research, our study found that infiltration of M2 macrophages was higher in cluster 2 with poor survival, suggesting that an increasing infiltration of M2 macrophage can be a risk factor for poor prognosis in BLCA.

Next, we observed the distribution of immune subtypes between the two clusters. The immune subtypes have six distinct immune subtypes, including Wound Healing (C1), IFN-gamma Dominant (C2), Inflammatory (C3), Lymphocyte Depleted (C4), Immunologically quiet (C5), and TGF-beta Dominant (C6). In our study, C2 IFN-g Dominant was particularly dominant in cluster 1, which presented a high CD8 T cell population and the greatest T-cell receptor (TCR) diversity (Soldevilla et al., 2019). However, cluster 2 had a higher proportion of C4 Lymphocyte Depleted than cluster 1, which could be associated with a high M2 macrophage response and indicate poor outcome (Soldevilla et al., 2019). These results above may explain the reason for the better prognosis of cluster 1.

We constructed a 10-gene risk model (PFKFB4, P4HB, OR2B6, OCIAD2, OAS1, KCNJ15, AHNAK, RAC3, EMP1, and PRKY) associated with the prognosis of BLCA patients. Researchers have demonstrated that higher expression of PFKFB4 is associated with poor prognosis and more frequent presence of metastases, including BLCA and other malignancies (Kotowski et al., 2021). High expression of P4HB has been observed in BLCA cell lines, knockdown of which can inhibit the invasion and proliferation of cancer cells (Lyu et al., 2020). Several studies have constructed prognosis models with OAS1, AHNAK, and other BLCA genes, which show the same trend as our analysis (Qiu et al., 2020; Guo et al., 2021; Jin et al., 2021). As for RAC3, one study has reported that it plays an oncogenic role to activate JAK/STAT signaling via up-regulation of PYCR1 in the BLCA cells (Cheng et al., 2020). Furthermore, we combined the TME prognostic signature with other clinicopathological prognostic factors to build a predictive nomogram model, which could offer an effective way for treatment planning and improving the overall outcome of BLCA patients. ROC curve and DCA confirmed that our model could accurately evaluate the prognosis of patients. When we compared our risk model with other prognosis risk signatures from Cao et al., Zhang et al., Yin et al. and Chen et al., the C-index demonstrated the highest AUC of our model. These results indicate that the overall performance of our proposed model is superior to others.

Considering the complexity between tumor growth and TME, we further explored the relationship between risk score and immune infiltration, and immune regulatory genes. We found that the risk score was negatively correlated with CD8^+^ T cell, Tfhs, and M1 macrophage, which were confirmed to play an anti-tumor role in cancer, while it was positively associated with M2 macrophage which inhibited the immune response. As a CD4^+^ T cell subset, Tfhs are essential for promoting humoral immune responses mediated by B cells, and can produce a negative or positive prognostic effect on multiple cancer types. A recent study has indicated that the accumulation of Tfh cells plays an important role in the CD8+-dependent antitumor immunity and anti-PD-L1 efficacy. The absence of Tfh cells is associated with the CD8^+^ T cell dysfunction, which leads to the resistance to anti-PD-L1 therapy (Niogret et al., 2021). Furthermore, during chronic infections and cancer, exposure to persistent antigens can lead to the state of T cells dysfunction, which is termed T cell exhaustion (Saeidi et al., 2018). Our results showed that the high-risk group with poor survival had a higher expression of T cell exhaustion markers, such as PDCD1/PD1, CTLA4, TIM3, LAG3, VSIR and TIGIT, which lead to tumor immune evasion.

Recent studies have shown that the high mutational burden of BLCA renders it susceptible to immunotherapy (Weinstein et al., 2014), particularly the immune checkpoint inhibitors. Unlike chemotherapy acting directly on cancerous tumors, immunotherapy works by enhancing the capacity of the immune system to fight against tumors, which has been approved to be the second-line treatment for advanced bladder cancer (Bellmunt et al., 2017). Besides the widely used PD-L1, MSI and TMB, we used two independent methods, IPS analysis and TIDE algorithm, to predict the response of TCGA-BLCA patients to ICIs. The results showed that IPS was significantly increased in the low-risk group while the TIDE score significantly decreased. And TIDE algorithm also indicated that the low-risk group appeared to present with a high rate of responder to ICIs immunotherapy. Both findings support the potential of the TME gene model to determine the immunotherapy sensitivity for TCGA-BLCA patients. IMvigor210 was a large phase 2 clinical trial in mUC that tested the efficacy and safety of atezolizumab which is the FDA approved PD-L1 inhibitor for BCa treatment. Notably, the performance of TME-related gene signature we developed was confirmed in mUC, which further demonstrated its value in predicting the immunotherapy response of BLCA patients.

There are also several limitations in our study. As this is a retrospective study based on the published data, the results should be further verified in prospective clinical trials. Moreover, the underlying mechanisms of how these TME-related genes in our risk model regulate the process of BLCA are still unclear. Their biological functions require further exploration with experiments.

## Conclusion

Our results demonstrated that TME-related gene signature shows potential roles in the prediction of prognosis and immunotherapy response in BLCA patients. In addition, the risk model is remarkably associated with immune cells infiltration and modulates the T cell function in BLCA, implying the potential role in predicting immunotherapy response. Therefore, the TME-related gene signature can provide recommendations for improving patients’ response to immunotherapy and promote personalized tumor immunotherapy in the future.

## Data Availability

The original contributions presented in the study are included in the article/Supplementary Material, further inquiries can be directed to the corresponding authors.

## Abbreviations

AUC, area under the curve; BLCA, bladder cancer; C-index, concordance index; CR, complete response; DCA, decision curve analysis; DCs, dendritic cells; DEGs, differentially expressed genes; ECM, extracellular matrix; GSEA, Gene Set Enrichment Analysis; HR, hazard ratio; iCAFs, inflammatory cancer-associated fibroblasts; ICIs, immune checkpoint inhibitors; IPS, immunophenoscore; MDSCs, myeloid-derived suppressor cells; MIBC, muscle-invasive bladder cancer; mUC: metastatic urothelial carcinoma; NMIBC: non-muscle invasive bladder cancer; OS: overall survival; PD, progressive disease; PFS, progression-free survival; PR, partial response; SD, stable disease; T regs, regulatory T cells; TAMs, tumor-associated macrophages; tDCs, tolerogenic dendritic cells; Tfhs, follicular helper T cells; TIDE, tumor immune dysfunction and exclusion; TME, tumor microenvironment.
