# Supplementary material for: Identification of tumor microenvironment-related signature for predicting prognosis and immunotherapy response in patients with bladder cancer
Source: Front Genet. 2022 Sep 6;13:923768. doi: 10.3389/fgene.2022.923768 (PMC9485450; doi:10.3389/fgene.2022.923768)
Supplement: Supplementary file 3 [file DataSheet1.pdf]

Supplementary Figures and Tables

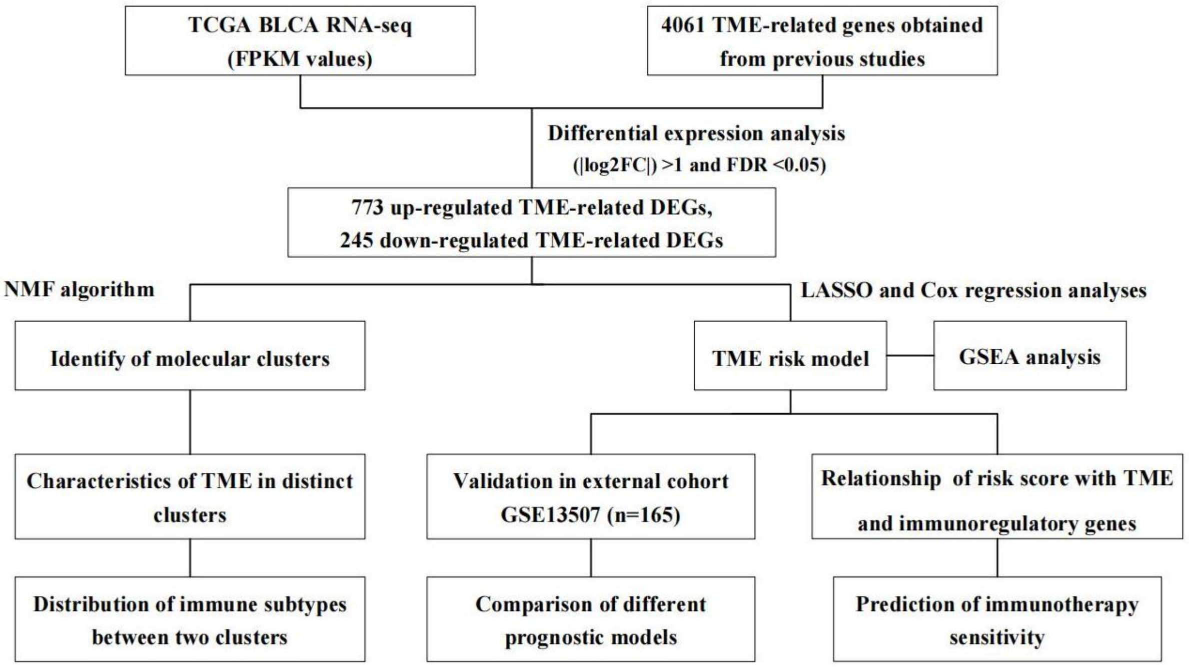

1.1 Supplementary Figures

Supplementary Figure 1. The entire analytical process of the study.

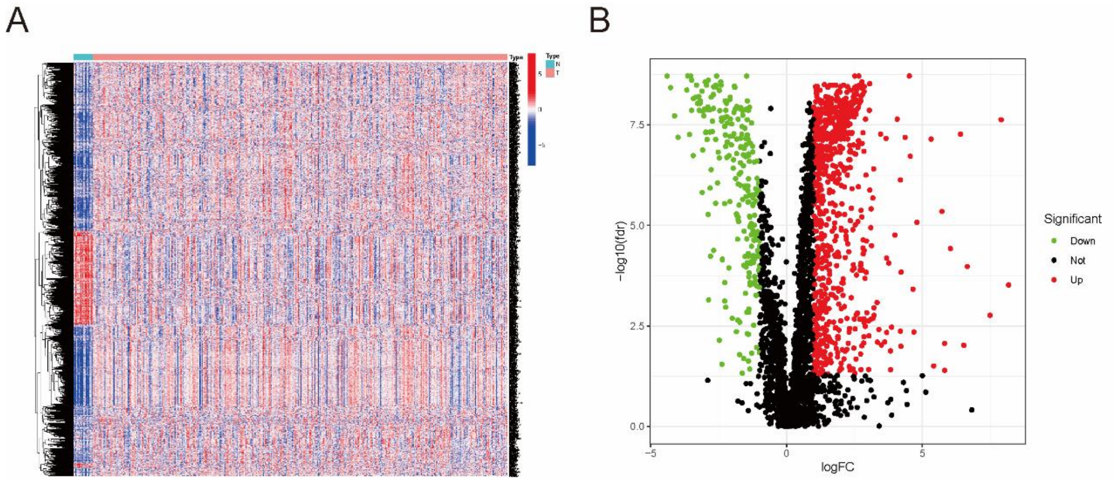

Supplementary Figure 2. Identification of differentially expressed TME-related genes in TCGA-BLCA cohort.

(A) Heatmap of TME-related DEGs. Blue represents downregulation, and red represents upregulation of genes. (B) Volcano plot of TME-related DEGs. Green dots represent downregulated genes; red dots represent upregulated genes.

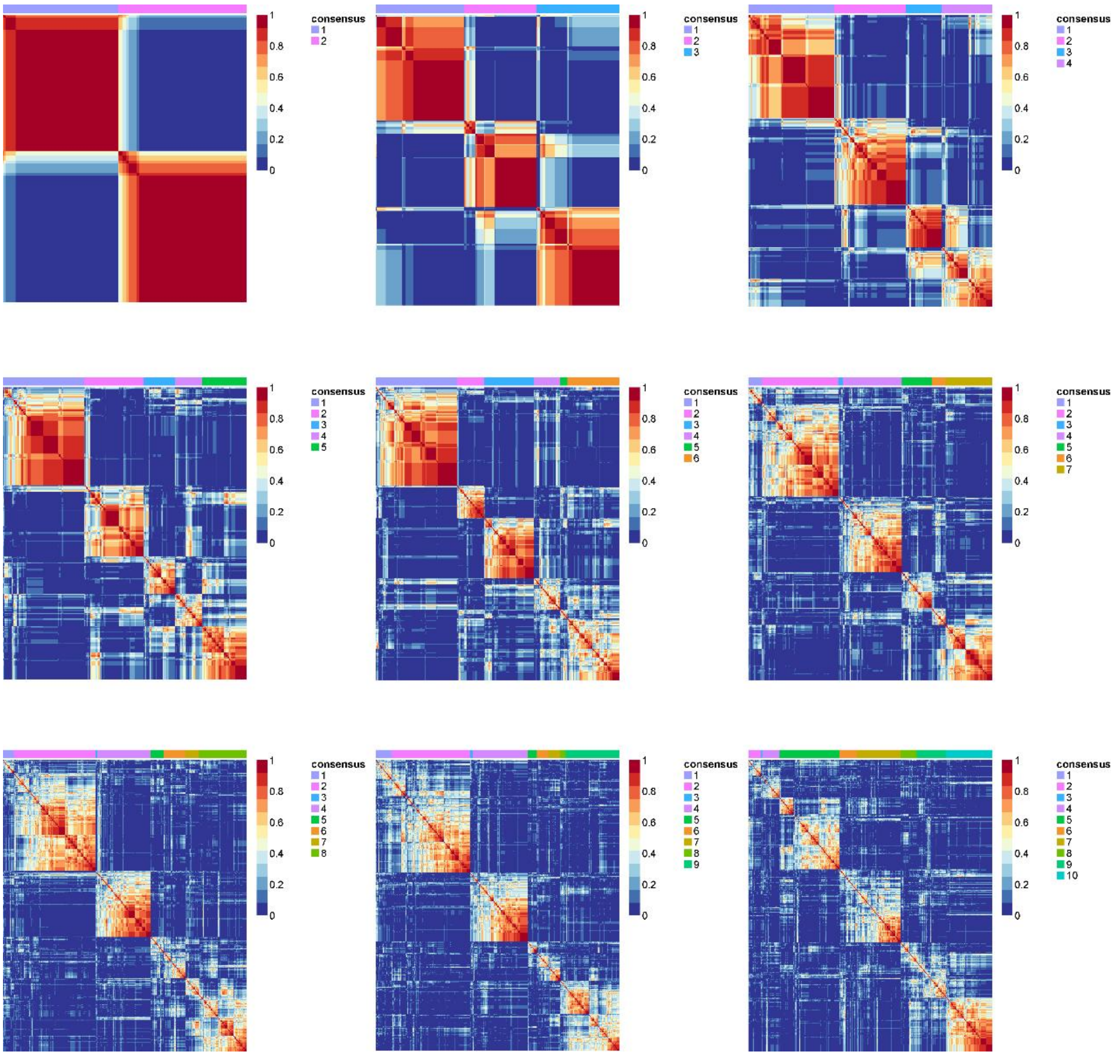

Supplementary Figure 3. Consensus map clustered via the NMF algorithm rank from 2-10.

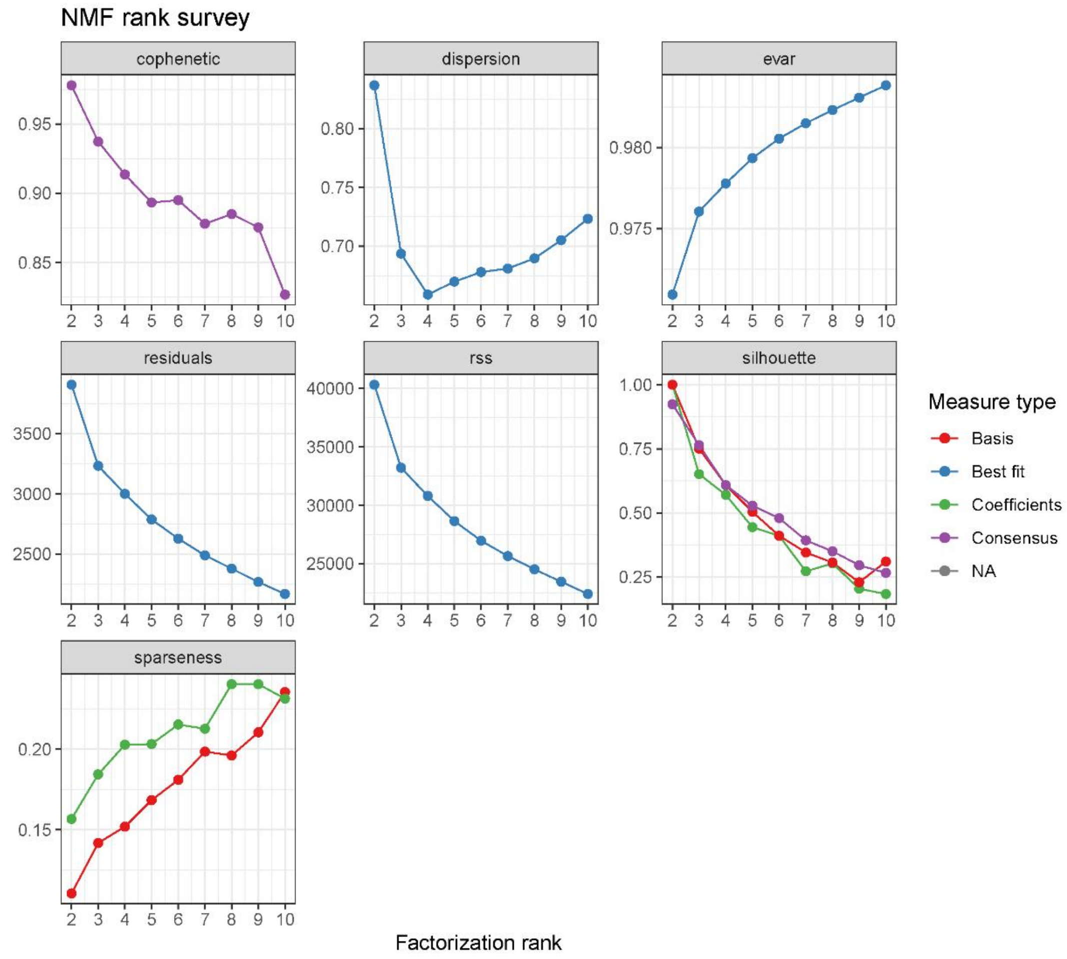

Supplementary Figure 4. NMF rank survey used to reflect the stability of the cluster.

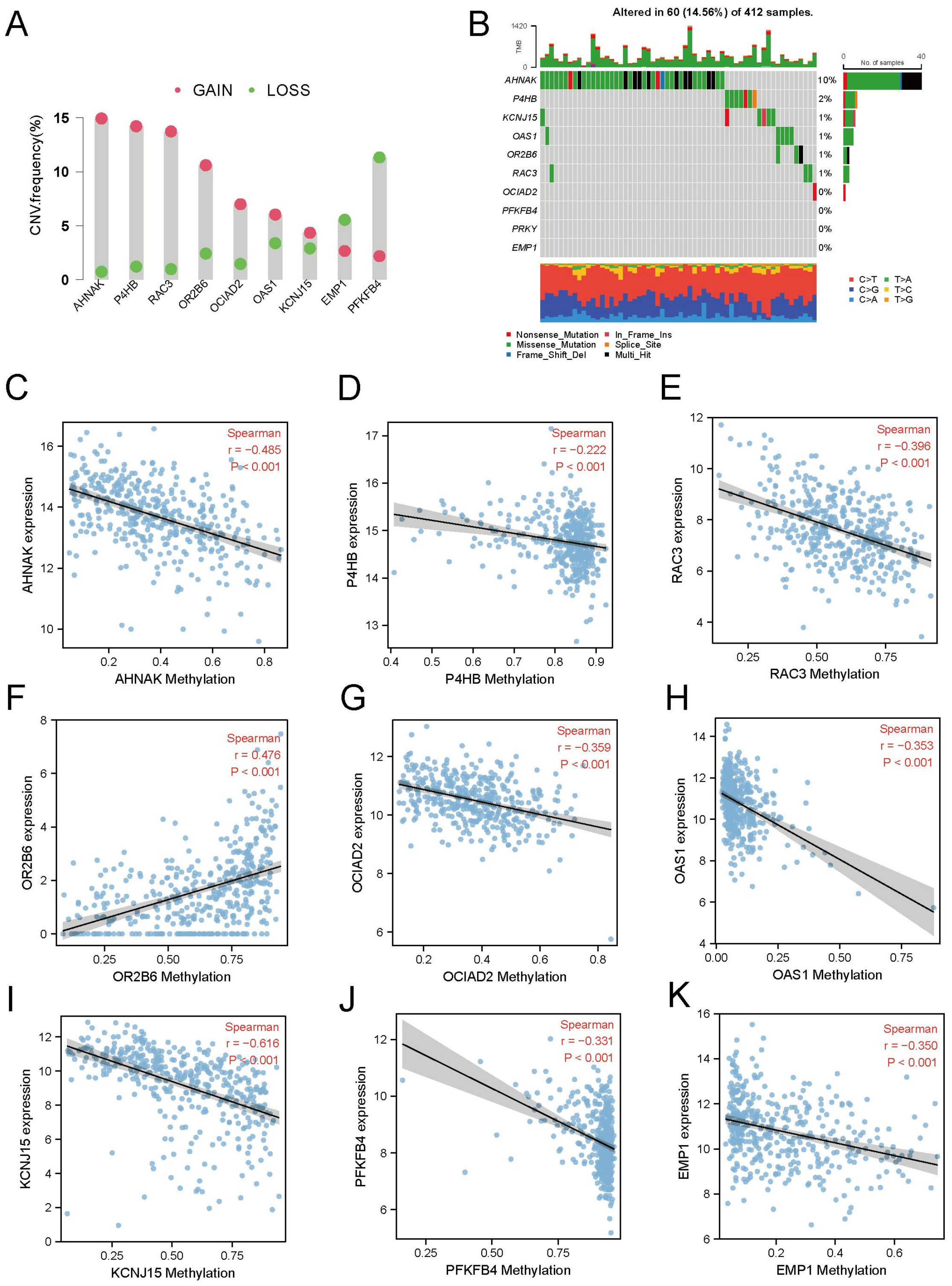

Supplementary Figure 5. The whole landscape of genetic alterations of TME-related genes in TCGA-BLCA cohort.

(A) The CNV variation frequency of TME-related genes. (B) The gene mutation frequency of TME-related genes. (C-K) Correlation analysis between the TME-related genes expression and DNA methylation level.

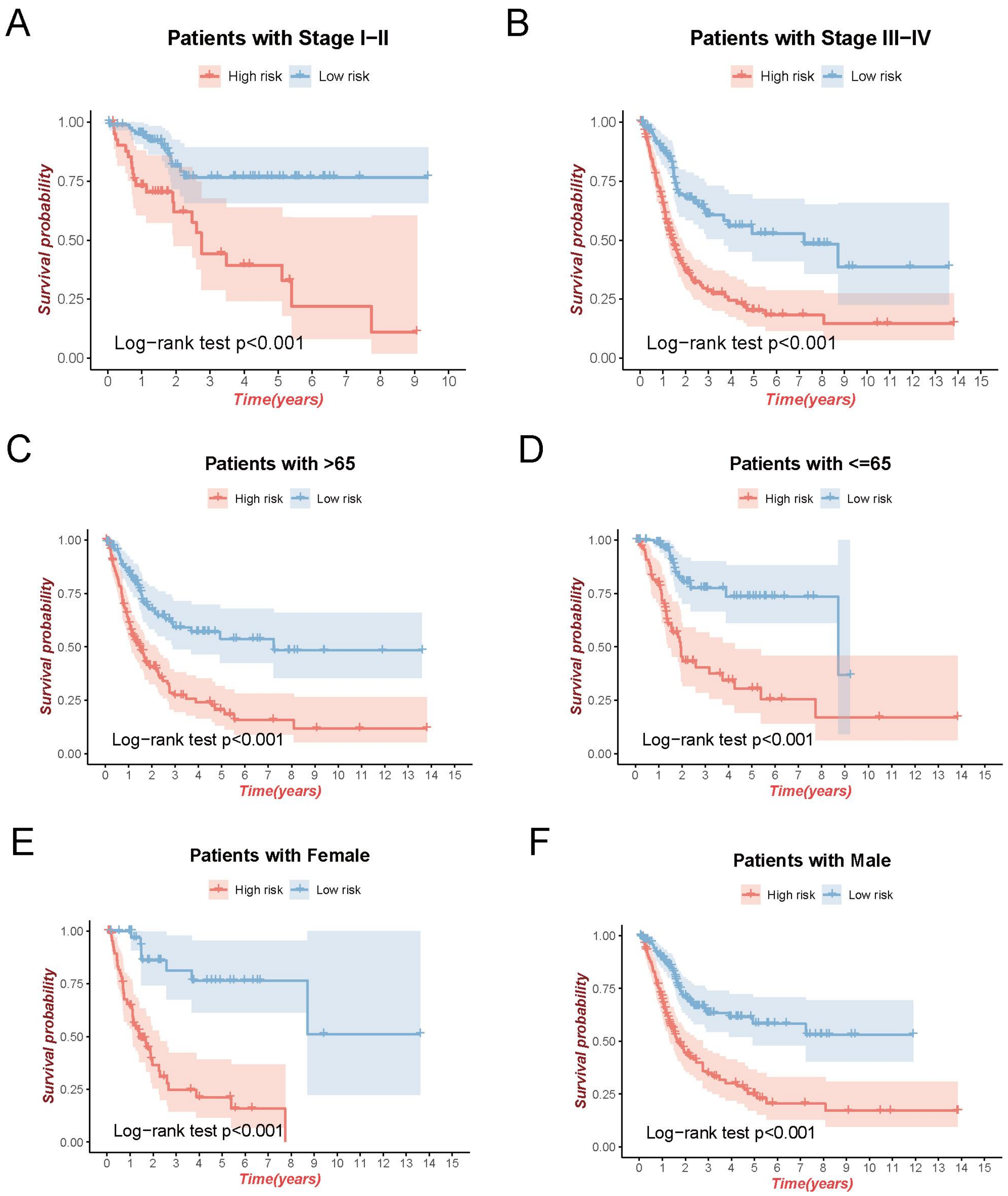

Supplementary Figure 6. Kaplan-Meier survival curves for patients with different clinical characteristics in TCGA-BLCA cohort.

(A) Patients with stage I-II. (B) Patients with stage III-IV. (C) Patients with age > 65. (D) Patients with age <= 65. (E) Patients with female; (F) Patients with male.

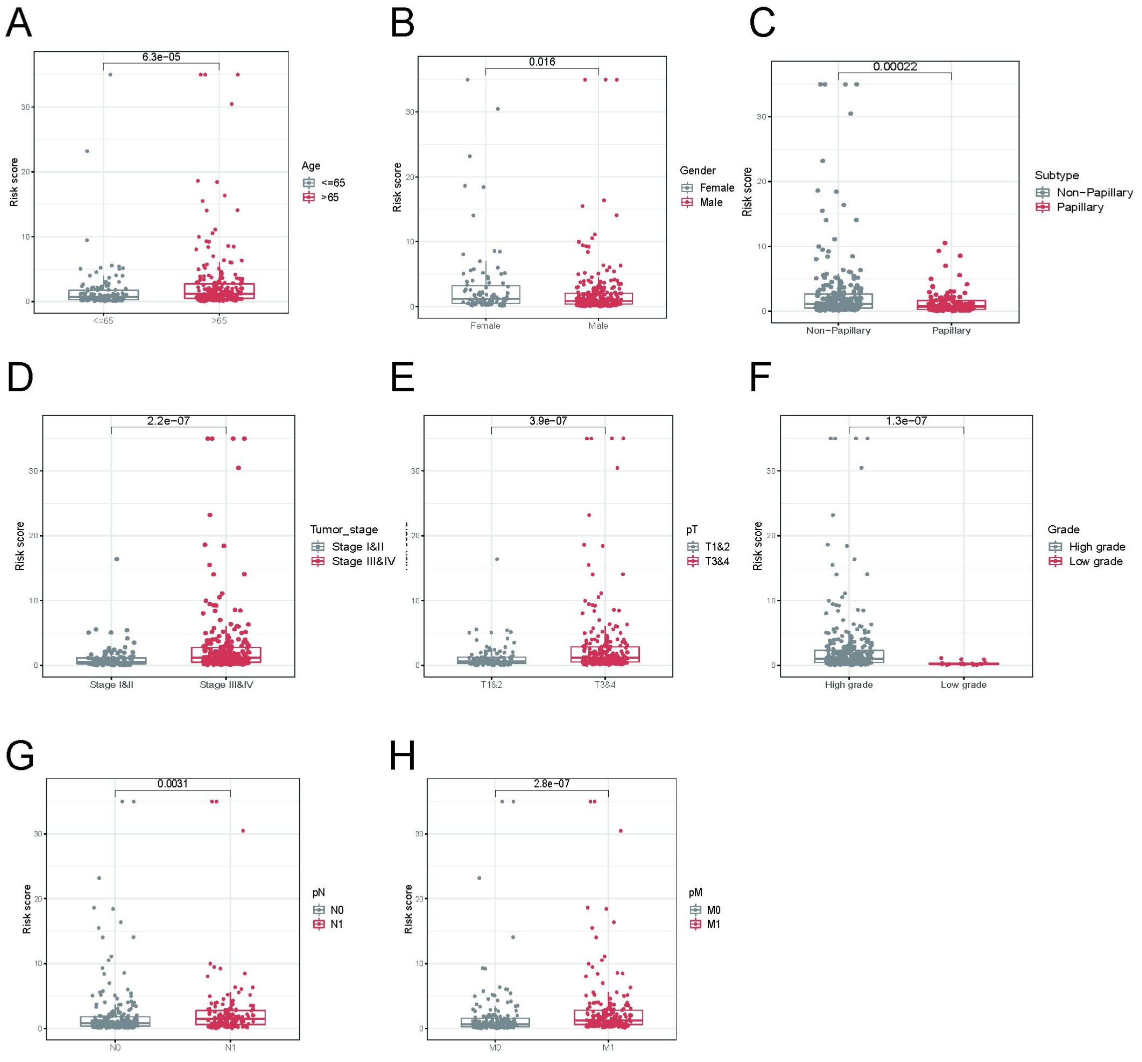

Supplementary Figure 7. The relationship between risk score and clinical characteristics in TCGA-BLCA cohort.

(A) Age (age <= 65 and age > 65 years old). (B) Gender (female and male). (C) Subtype (Non-papillary and papillary). (D) Tumor stage (I&II or III&IV). (E) pT (T1&2 or T3&4). (F) Grade (high grade and low grade). (G) pN (N0 and N1). (H) pM (M0 and M1).

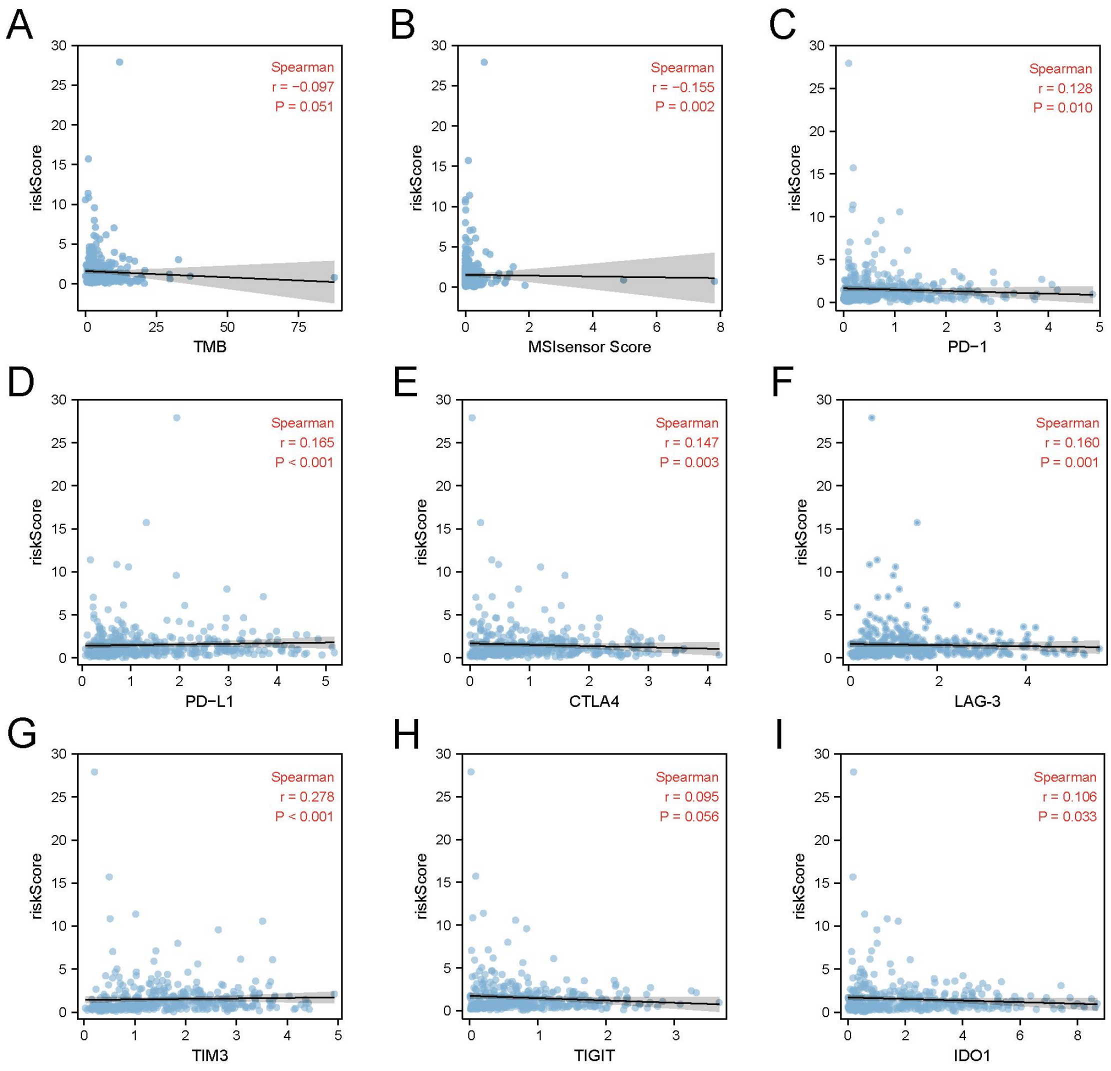

Supplementary Figure 8. Correlation analysis between risk score, TMB , MSIsensor Score, and several classical immune checkpoint.

(A) Correlation analysis between the risk score and TMB. (B) Correlation analysis between the risk score and MSIsensor Score. (C-I). Scatter plots of risk score and several classical immune checkpoint.

1.2 Supplementary Tables

Supplementary Table 1. 4061 TME-related genes.

Supplementary Table 2. 1018 differentially expressed TME-related genes in the TCGA-BLCA cohort.

Supplementary Table 3. Details of GSEA results in high-risk and low-risk groups.
